# Supplementary material for: Incidence, risk factors and healthcare costs of central line-associated nosocomial bloodstream infections in hematologic and oncologic patients
Source: PLoS One. 2020 Jan 24;15(1):e0227772. doi: 10.1371/journal.pone.0227772 (PMC6980604; doi:10.1371/journal.pone.0227772)
Supplement: S1 Table — For patients with central line-associated bloodstream infections (CLABSI) the time at risk (admission to onset of CLABSI) is used, for the non-CLABSI patients the time from admission to discharge or death is used. (DOCX) [file pone.0227772.s001.docx]

All items included in the univariable analysis. For patients with central line-associated bloodstream infections (CLABSI) the time at risk (admission to onset of CLABSI) is used, for the non-CLABSI patients the time from admission to discharge or death is used.

| **Influencing factors** | | **No. of patients with CLABSI** | | **No. of patients without CLABSI** | | **RR*** | | **95%CI**** | | | | | **p-value***** |
| --- | --- | --- | --- | --- | --- | --- | --- | --- | --- | --- | --- | --- | --- |
| **Total** | | 111 | | 499 | |  | |  | | | | |  |
| **Age in years** | | | | | | | | | | | | | |
| >50 (Median) | | 64 | | 224 | | 1.52 | | 1.08 | | - | 2.14 | | 0.016 |
| <41 | | 29 | | 177 | | 0.69 | | 0.47 | | - | 1.02 | | 0.060 |
| 41 – 50 | | 18 | | 98 | | 0.82 | | 0.52 | | - | 1.31 | | 0.504 |
| 51 – 60 | | 36 | | 115 | | 1.46 | | 1.03 | | - | 2.08 | | 0.051 |
| ≥61 | | 28 | | 109 | | 1.16 | | 0.79 | | - | 1.71 | | 0.452 |
| **Gender** | | | | | | | | | | | | | |
| Male | | 69 | | 306 | | 1.03 | | 0.73 | | - | 1.46 | | 0.914 |
| **Underlying diseases** | | | | | | | | | | | | | |
| Acute myeloid leukemia | | 72 | | 157 | | 3.07 | | 2.16 | | - | 4.37 | | <0.001 |
| Non Hodgkin Lymphoma | | 18 | | 179 | | 0.41 | | 0.25 | | - | 0.65 | | <0.001 |
| Acute lymphoid leukemia | | 10 | | 63 | | 0.73 | | 0.40 | | - | 1.33 | | 0.335 |
| Malignancy of the testicles | | 1 | | 43 | | 0.12 | | 0.02 | | - | 0.82 | | 0.002 |
| Chronic myeloid leukemia | | 3 | | 4 | | 2.39 | | 1.00 | | - | 5.73 | | 0.117 |
| Hodgkin Lymphoma | | 1 | | 14 | | 0.36 | | 0.05 | | - | 2.41 | | 0.328 |
| Others | | 6 | | 39 | | 0.72 | | 0.33 | | - | 1.54 | | 0.546 |
| **Comorbidities** | | | | | | | | | | | | | |
| Cardiac disease | | 37 | | 110 | | 1.57 | | 1.11 | | - | 2.23 | | 0.014 |
| Pulmonary disease | | 19 | | 55 | | 1.50 | | 0.97 | | - | 2.30 | | 0.079 |
| Kidney disease | | 23 | | 71 | | 1.43 | | 0.96 | | - | 2.15 | | 0.109 |
| Gastrointestinal disease | | 21 | | 87 | | 1.08 | | 0.71 | | - | 1.66 | | 0.682 |
| Smoking | | 15 | | 86 | | 0.79 | | 0.48 | | - | 1.30 | | 0.398 |
| Neurologic disease | | 11 | | 38 | | 1.26 | | 0.73 | | - | 2.18 | | 0.440 |
| Endocrine disorder | | 8 | | 29 | | 1.20 | | 0.64 | | - | 2.28 | | 0.518 |
| Trauma | | 5 | | 29 | | 0.80 | | 0.35 | | - | 1.82 | | 0.819 |
| Diabetes mellitus | | 6 | | 26 | | 1.03 | | 0.49 | | - | 2.17 | | 1.000 |
| Other malignancies | | 7 | | 35 | | 0.91 | | 0.45 | | - | 1.83 | | 1.000 |
| Other diseases | | 34 | | 117 | | 1.34 | | 0.94 | | - | 1.92 | | 0.116 |
| **Weight (body mass index, kg/m^2^)** | | | | | | | | | | | | | |
| >25 (Median) | | 51 | | 224 | | 1.04 | | 0.74 | |  | 1.45 | | 0.916 |
| < 18.5 | | 3 | | 36 | | 0.41 | | 0.14 | | - | 1.22 | | 0.088 |
| 18.5 – 24.9 | | 57 | | 235 | | 1.15 | | 0.82 | | - | 1.61 | | 0.462 |
| 25 – 29.9 | | 30 | | 169 | | 0.76 | | 0.52 | | - | 1.12 | | 0.180 |
| > 30 | | 21 | | 59 | | 1.55 | | 1.02 | | - | 2.34 | | 0.061 |
| **Length of hospital stay in days** | | | | | | | | | | | | | |
| > 18 (Median) | | 32 | | 258 | | 0.45 | | 0.31 | | - | 0.65 | | <0.001 |
| <14 | | 44 | | 196 | | 1.01 | | 0.72 | | - | 1.43 | | 1 |
| 14 – 27 | | 53 | | 145 | | 1.90 | | 1.37 | | - | 2.65 | | <0.001 |
| 28 – 41 | | 3 | | 107 | | 0.13 | | 0.04 | | - | 0.39 | | <0.001 |
| 42 – 55 | | 5 | | 26 | | 0.88 | | 0.39 | | - | 2.00 | | 1.000 |
| 56 – 69 | | 3 | | 14 | | 0.97 | | 0.34 | | - | 2.74 | | 1.000 |
| >69 | | 3 | | 11 | | 1.18 | | 0.43 | | - | 3.27 | | 0.727 |
| **Length of chemotherapy in days** | | | | | | | | | | | | | |
| <5 (Median) | | 26 | | 264 | | 0.34 | | 0.22 | | - | 0.51 | | <0.001 |
| 5 – 9 | | 59 | | 137 | | 2.06 | | 1.50 | | - | 2.82 | | <0.001 |
| 10 – 19 | | 14 | | 21 | | 2.37 | | 1.52 | | - | 3.70 | | 0.002 |
| 20 – 29 | | 5 | | 41 | | 0.58 | | 0.25 | | - | 1.35 | | 0.233 |
| ≥30 | | 7 | | 31 | | 1.01 | | 0.51 | | - | 2.02 | | 1.000 |
| **Chemotherapy agents** | | | | | | | | | | | | | |
| Antimetabolites | | 92 | | 318 | | 2.36 | | 1.49 | | - | 3.76 | | <0.001 |
| Alkylating agents | | 68 | | 272 | | 1.26 | | 0.89 | | - | 1.78 | | 0.206 |
| Alkaloids | | 62 | | 284 | | 0.97 | | 0.69 | | - | 1.35 | | 0.833 |
| Antibiotic agents with cytostatic effect | | 32 | | 169 | | 0.82 | | 0.57 | | - | 1.20 | | 0.318 |
| Monoclonal antibodies | | 1 | | 67 | | 0.07 | | 0.01 | | - | 0.51 | | <0.001 |
| Tyrosinkinase inhibitors | | 1 | | 3 | | 1.38 | | 0.25 | | - | 7.58 | | 0.553 |
| Other chemotherapeutic agents | | 96 | | 451 | | 0.74 | | 0.46 | | - | 1.19 | | 0.229 |
| Other immunosuppressive agents | | 66 | | 216 | | 1.71 | | 1.21 | | - | 2.41 | | 0.002 |
| **Antibiotic therapy and antibiotic agents** | | | | | | | | | | | | | |
| Fluoroquinolones | | 12 | | 410 | | 0.05 | | 0.03 | | - | 0.10 | | <0.001 |
| Antifungal agents | | 91 | | 346 | | 1.80 | | 1.15 | | - | 2.83 | | 0.007 |
| Broad spectrum penicillins | | 65 | | 166 | | 2.32 | | 1.65 | | - | 3.26 | | <0.001 |
| Betalactamase inhibitors | | 65 | | 165 | | 2.33 | | 1.66 | | - | 3.28 | | <0.001 |
| Aminoglycosides | | 41 | | 61 | | 2.92 | | 2.12 | | - | 4.02 | | <0.001 |
| Carbapenems | | 32 | | 75 | | 1.90 | | 1.34 | | - | 2.71 | | 0.001 |
| Glycopeptides | | 10 | | 76 | | 0.60 | | 0.33 | | - | 1.11 | | 0.098 |
| Nitroimidazoles | | 17 | | 54 | | 1.37 | | 0.87 | | - | 2.16 | | 0.191 |
| Lincosamides | | 6 | | 26 | | 1.03 | | 0.49 | | - | 2.17 | | 1.000 |
| Cephalosporins | | 8 | | 13 | | 2.18 | | 1.23 | | - | 3.86 | | 0.037 |
| Others | | 3 | | 11 | | 1.18 | | 0.43 | | - | 3.27 | | 0.727 |
| **Transplantation** | | | | | | | | | | | | | |
| Any transplantation | | 47 | | 125 | | 1.87 | | 1.34 | | - | 2.61 | | <0.001 |
| Allogenic HSCT | | 32 | | 70 | | 2.02 | | 1.42 | | - | 2.87 | | <0.001 |
| Autologous HSCT | | 14 | | 52 | | 1.19 | | 0.72 | | - | 1.96 | | 0.501 |
| Allogenic BMT | | 1 | | 3 | | 1.38 | | 0.25 | | - | 7.58 | | 0.553 |
| **Transfusion** | | | | | | | | | | | | | |
| Any transfusion | | 72 | | 325 | | 0.99 | | 0.70 | | - | 1.41 | | 1.000 |
| Erythrocyte transfusion | | 27 | | 286 | | 0.31 | | 0.20 | | - | 0.46 | | <0.001 |
| Thrombocyte transfusion | | 68 | | 270 | | 1.27 | | 0.90 | | - | 1.80 | | 0.205 |
| Others | | 3 | | 34 | | 0.43 | | 0.14 | | - | 1.29 | | 0.124 |
| **Parenteral nutrition** | | | | | | | | | | | | | |
| Parenteral nutrition | | 3 | | 7 | | 1.67 | | 0.64 | | - | 4.36 | | 0.400 |
| **Anemia** | | | | | | | | | | | | | |
| Anemia | | 108 | | 336 | | 13.46 | | 4.33 | | - | 41.80 | | <0.001 |
| **Leukocyte count** | | | | | | | | | | | | | |
| 3,000 – 3,999/µL | | 0 | | 45 | | 0.00 | | Not defined | | | | | <0.001 |
| 2,000 – 2,999/µL | | 0 | | 43 | | 0.00 | | Not defined | | | | | <0.001 |
| 1,000 – 1,999/µL | | 2 | | 46 | | 0.21 | | 0.05 | | - | 0.84 | | 0.006 |
| <1,000/µL | | 109 | | 279 | | 31.18 | | 7.78 | | - | 125.05 | | <0.001 |
| **Duration of leukocytopenia in days** | | | | | | | | | | | | | |
| >12 (Median) | | 56 | | 230 | | 1.15 | | 0.82 | | - | 1.61 | | 0.462 |
| <4 | | 1 | | 160 | | 0.03 | | 0.00 | | - | 0.18 | | <0.001 |
| 4 – 13 | | 63 | | 120 | | 3.06 | | 2.20 | | - | 4.27 | | <0.001 |
| 14 – 23 | | 29 | | 100 | | 1.32 | | 0.91 | | - | 1.92 | | 0.159 |
| ≥24 | | 18 | | 119 | | 0.67 | | 0.42 | | - | 1.07 | | 0.101 |
| **Thrombocytopenia** | | | | | | | | | | | | | |
|  | <100.000/µL | | 109 | | 326 | 21.93 | 5.48 | | **-** | | | 87.81 | <0.001 |
| **Indication for CVC insertion** | | | | | | | | | | | | | |
| Planned chemotherapy | | 60 | | 403 | | 0.37 | | 0.27 | | - | 0.52 | | <0.001 |
| Planned conditioning | | 44 | | 67 | | 2.95 | | 2.14 | | - | 4.07 | | <0.001 |
| CVC change due to local infection | | 1 | | 11 | | 0.45 | | 0.07 | | - | 2.98 | | 0.704 |
| Planned total parenteral nutrition | | 2 | | 7 | | 1.23 | | 0.36 | | - | 4.21 | | 0.671 |
| Not specified | | 5 | | 21 | | 1.06 | | 0.47 | | - | 2.37 | | 0.800 |
| **Number of CVC inserted** | | | | | | | | | | | | | |
| >1 CVC inserted | | 38 | | 28 | | 4.29 | | 3.19 | | - | 5.78 | | <0.001 |
| **Place in the hospital where the CVC was inserted** | | | | | | | | | | | | | |
| Ambulance room | | 104 | | 458 | | 1.27 | | 0.63 | | - | 2.57 | | 0.696 |
| Intensive care unit | | 7 | | 37 | | 0.87 | | 0.43 | | - | 1.75 | | 0.840 |
| Other | | 0 | | 3 | | 0.00 | | Not defined | | | | | 1.000 |
| **Level of experience of the anesthesiologist who inserted the CVC** | | | | | | | | | | | | | |
| Assistant physician | | 42 | | 229 | | 0.76 | | 0.54 | | - | 1.08 | | 0.139 |
| Senior physician | | 48 | | 200 | | 1.11 | | 0.79 | | - | 1.56 | | 0.593 |
| Consultant | | 13 | | 43 | | 1.31 | | 0.79 | | - | 2.18 | | 0.362 |
| Other training level | | 0 | | 2 | | 0 | | Not defined | | | | | 1.000 |
| **CVC dressing** | | | | | | | | | | | | | |
| Antiseptic dressing | | 40 | | 170 | | 1.07 | | 0.76 | | - | 1.52 | | 0.741 |
| **CVC insertion site** | | | | | | | | | | | | | |
| **V. basilica** | | 1 | | 3 | | 1.38 | | 0.25 | | - | 7.58 | | 0.553 |
| V. basilica, right | | 1 | | 1 | | 2.76 | | 0.68 | | - | 11.17 | | 0.331 |
| V. basilica, left | | 0 | | 2 | | 0.00 | | Not defined | | | | | 1.000 |
| **V. jugularis** | | 95 | | 372 | | 1.82 | | 1.11 | | - | 2.98 | | 0.013 |
| V. jugularis, right | | 76 | | 301 | | 1.34 | | 0.93 | | - | 1.93 | | 0.130 |
| V. jugularis, left | | 19 | | 82 | | 1.04 | | 0.67 | | - | 1.62 | | 0.888 |
| **V. subclavia** | | 15 | | 132 | | 0.49 | | 0.30 | | - | 0.82 | | 0.003 |
| V. subclavia, right | | 11 | | 81 | | 0.62 | | 0.35 | | - | 1.11 | | 0.107 |
| V. subclavia, left | | 4 | | 56 | | 0.34 | | 0.13 | | - | 0.90 | | 0.013 |
| **V. femoralis** | | 0 | | 2 | | 0.00 | | Not defined | | | | | 1.000 |
| V. femoralis, right | | 0 | | 2 | | 0.00 | | Not defined | | | | | 1.000 |
| **Length of CVC usage in days** | | | | | | | | | | | | | |
| >12 (Median) | | 52 | | 237 | | 0.98 | | 0.70 | | - | 1.37 | | 0.917 |
| <8 | | 13 | | 203 | | 0.24 | | 0.14 | | - | 0.42 | | <0.001 |
| 8 – 14 | | 60 | | 85 | | 3.77 | | 2.73 | | - | 5.21 | | <0.001 |
| 15 – 21 | | 20 | | 110 | | 0.81 | | 0.52 | | - | 1.26 | | 0.373 |
| >22 | | 18 | | 101 | | 0.80 | | 0.50 | | - | 1.27 | | 0.358 |

*Risk ratio. ** 95%-confidence interval. ***Fisher’s Exact test (2-tailed P). CLABSI = central line-associated bloodstream infection. CVC = central venous catheter. HSCT = hematopoietic stem cell transplantation. BMT = bone marrow transplantation.
