# Supplementary material for: Incidence, risk factors and healthcare costs of central line-associated nosocomial bloodstream infections in hematologic and oncologic patients
Source: PLoS One. 2020 Jan 24;15(1):e0227772. doi: 10.1371/journal.pone.0227772 (PMC6980604; doi:10.1371/journal.pone.0227772)
Supplement: S2 Table — Selected characteristics of the case patients and the control patients. For patients with central line-associated bloodstream infections (CLABSI) the time at risk (admission to onset of CLABSI) is used, for the non-CLABSI patients the time from admission to discharge or death is used. (DOCX) [file pone.0227772.s002.docx]

|  | **Case patients with CLABSI**  **(n=79)** | **Control patients without CLABSI**  **(n=158)** | **p-value*** |
| --- | --- | --- | --- |
| **Median age (years)** | 52 | 53 | 0.661^a^ |
| **Male** | 47 | 83 | 0.335 |
| **Deceased patients** | 3 | 2 | 0.337 |
| **Median length of CVC usage (days)** | 12 | 22 | <0.001^a^ |
| **Medial length of hospital stay (days)** | 15 | 30 | <0.001^a^ |

**Cost analysis.** Selected characteristics of the case patients and the control patients. For patients with central line-associated bloodstream infections (CLABSI) the time at risk (admission to onset of CLABSI) is used, for the non-CLABSI patients the time from admission to discharge or death is used.

Data are No. of patients unless otherwise indicated.

*p-value, Fisher’s Exact test for binary parameters and Chi-square test for parameters with >2 categories

^a^Wilcoxon rank sum test

CLABSI=Central line-associated bloodstream infection
